# Risk Factors for Antibiotic Exposure Post–Fecal Microbiota Transplantation for Recurrent *Clostridioides difficile* Infection: A Prospective Multicenter Observational Study

**DOI:** 10.1093/ofid/ofaf130

**Published:** 2025-03-07

**Authors:** William Hirsch, Monika Fischer, Alexander Khoruts, Jessica R Allegretti, Colleen R Kelly, Byron Vaughn

**Affiliations:** Department of Medicine, University of Minnesota, Minneapolis, Minnesota, USA; Division of Gastroenterology and Hepatology, Department of Medicine, Indiana University, Indianapolis, Indiana, USA; Division of Gastroenterology, Hepatology, and Nutrition, Department of Medicine, University of Minnesota, Minneapolis, Minnesota, USA; Center for Immunology, University of Minnesota, Minneapolis, Minnesota, USA; BioTechnology Institute, University of Minnesota, St Paul, Minnesota, USA; BioTechnology Institute, University of Minnesota, St Paul, Minnesota, USA; Division of Gastroenterology, Brigham and Women's Hospital, Harvard Medical School, Boston, Massachusetts, USA; Division of Gastroenterology, Hepatology, and Nutrition, Department of Medicine, University of Minnesota, Minneapolis, Minnesota, USA

**Keywords:** antimicrobial use, *Clostridium difficile* infection, fecal microbiota transplantation, recurrent *Clostridioides difficile* infection, antimicrobial stewardship

## Abstract

**Background:**

Recurrent *Clostridioides difficile* infection (CDI) is primarily driven by antibiotic-induced disruption of the indigenous intestinal microbiota. Restoration of microbiota through fecal microbiota transplantation (FMT) is effective in preventing subsequent CDI, although this effect is attenuated with additional antibiotic exposure. The aim of this study was to identify the risk factors for recurrent antibiotic administration after FMT.

**Methods:**

This is a prospective cohort of patients who were administered FMT for recurrent CDI from 1 July 2019 through 23 November 2023 across 6 institutions in the United States. Providers collected de-identified data at the time of FMT administration and in the months post–FMT administration.

**Results:**

The analysis included 448 patients. Risk factors for non-CDI antibiotic administration within 2 months of FMT included immunocompromised status (odds ratio [OR], 2.2 [95% confidence interval {CI}, 1.1–4.4]; *P* = .02), >3 non-CDI antibiotic courses pre-FMT (OR, 3.1 [95% CI, 1.4–6.8]; *P* = .006), and prior hospitalization for CDI (OR, 2.0 [95% CI, 1.1–3.8]; *P* = .02). The most common indications for non-CDI antibiotic administration post-FMT were urinary tract infections, respiratory infections, and procedure prophylaxis.

**Conclusions:**

Non-CDI antibiotic exposure significantly increases the risk of CDI recurrence post-FMT. Risk factors for non-CDI antibiotic administration within 2 months of FMT include immunocompromised status, multiple prior non-CDI antibiotics, and prior hospitalization for CDI. These individuals may benefit from additional or modified recurrent CDI prevention strategies.

*Clostridioides difficile* is an anaerobic gram-positive spore-forming bacterium that causes infection in the colon typically following recent antibiotic exposure. After an initial infection, recurrent *C difficile* infection (rCDI) occurs in approximately 20% of patients with higher rates of recurrence and is associated with subsequent episodes [1–4]. Additional courses of antibiotic administration perpetuate intestinal dysbiosis, leading to a cycle of rCDI. Fecal microbiota transplantation (FMT) is effective at breaking the cycle of rCDI [5–14] and is endorsed by multiple society guidelines for the management of rCDI [15, 16].

The pathophysiology of *C difficile* infection (CDI) involves progressive intestinal dysbiosis with loss of colonization resistance, the inherent property of a healthy microbiota to resist infection. Most individuals develop CDI initially due to antibiotic-induced intestinal dysbiosis [17]. However, other known or potential risk factors for CDI include older age, immunosuppressed status, cognitive impairment, proton pump inhibitor use, inflammatory bowel disease, and renal dysfunction [14, 18–25]. It is unknown if these are risk factors for dysbiosis, *C difficile* exposure, or both. Although FMT is effective in ameliorating dysbiosis and preventing CDI recurrence, antibiotic administration in the first 8 weeks post-FMT may limit the ability to effectively restore a healthy colonic microbial community structure and thus contribute to CDI recurrence post-FMT [14, 26]. Although there are scant data, approximately 10% of patients may receive additional non-CDI antibiotics over this time [26]. Little is known about risk factors for antibiotic exposure in this critical timeframe. Identification of individuals likely to receive antibiotic therapy post-FMT could lead providers to consider alternative strategies to mitigate rCDI. The aims of this study are to describe the association of non-CDI antibiotic use post-FMT and to identify patient characteristics at the time of FMT that predict subsequent non-CDI antibiotic exposure.

## METHODS

This is a secondary analysis of a prospective cohort study of patients treated for rCDI with FMT from 1 July 2019 through 23 November 2023. No formal sample size was calculated for the cohort as the initial intent was to understand and explore the efficacy and safety of FMT in a real-world setting. The cohort methodology, including preparation of FMT product, inclusion/exclusion criteria, and administration protocols followed by participating institutions, is published in an earlier version of this cohort (through 28 October 2021) aimed at assessing the impact of FMT route on effectiveness [14]. No changes to the methods occurred since the prior publication. In brief, 6 centers were supplied liquid or capsule formulations of standardized FMT material for colonoscopic or oral administration, respectively. FMT product was provided without charge to the participating institutions with the requirement of collecting and reporting de-identified demographic and outcomes data for treated patients. Participating institutions included 4 academic centers and 2 community gastroenterology groups. Data were entered into a Research Electronic Data Capture (REDCap) database maintained by the University of Minnesota. Consent for FMT was performed by the treating physician based on current United States Food and Drug Administration (FDA) guidance. Because no personal health information was collected, a waiver of consent was obtained, although local institutional review boards at some sites required consent to share de-identified data. Demographic and clinical characteristics were added by the clinical care team from medical record review and interview with the patient. The protocol was approved by the University of Minnesota Institutional Review Board.

### Data Collection

Data on CDI recurrence and non-CDI antibiotic administration were collected after follow-up visits with the treating institution at the following intervals: 1 month, 2 months, 6 months, and 12 months. However, specific dates were not included. Providers were encouraged to obtain data from a patient encounter (phone call or visit). Automated survey reminders were sent to the treating physician prior to the scheduled follow-up evaluations. In the absence of a clinical encounter, providers were requested to extract outcome data from their institutional electronic medical record.

From the cohort of patients who received FMT, those with both follow-up data on CDI outcomes at 2 months and non-CDI antibiotics at 2 months were included in the final analysis set for the main outcome. Individuals who experienced a CDI recurrence at 1 month had their CDI recurrence and non-CDI antibiotic exposure imputed forward to 2 months. Patients who were administered FMT for an indication of severe or fulminant were excluded as this represents a distinct disease from prevention of recurrence CDI.

### Outcome Definition

The primary outcome was non-CDI antibiotic exposure 2 months after FMT. Non-CDI antibiotics exposure was determined by the treating physician. Providers had the option of free texting the antibiotic administered and the indication. CDI recurrence was determined by both positive laboratory testing for CDI based on local practices and physician initiation of an anti-CDI antibiotic. Both components were required to assess an individual as CDI recurrence. Anti-CDI antibiotics included vancomycin, fidaxomicin, or metronidazole.

### Risk Factor Definitions

Potential risk factors for non-CDI antibiotic exposure were assessed including baseline demographics, CDI disease history, and other notable comorbidities including immunocompromised status and neuromuscular impairment. Patients were defined as being immunocompromised if they had either a primary immunodeficiency or secondary immunodeficiency from medication including prednisone >10 mg/day [27], immunomodulator medication use, any biologic therapy, calcineurin inhibitors, ongoing cancer therapy, or other medication or condition deemed by the treating provider to compromise the immune system. Comorbid conditions such as chronic kidney disease requiring hemodialysis, decompensated end-stage liver disease, cognitive impairment, and inflammatory bowel disease were reported by the treating provider based on medical record review. Neuromuscular impairment was defined through the opinion of the provider as a condition that could impair normal mobility, such as getting to the bathroom. FMT route was documented; however, only oral and colonoscopy administered were included in regression analysis.

### Statistical Analysis

Demographic data are summarized as counts and percentages. When applicable, data are reported as median with associated interquartile range (IQR). Significance among predictor variables and the outcomes of recurrence was determined by Student *t* test or Wilcoxon test for continuous variables (as appropriate based on normality) and with Fisher exact test for categorical variables. Exploratory univariate logistic regression was performed to examine non-CDI antibiotic exposure as a risk for CDI recurrence within 2 months and to identify potential risk factors associated non-CDI antibiotic exposure within 2 months of FMT administration. In both cases, age and sex were included into the multivariate model, along with any other variable found to be significant on univariate analysis. Statistical analysis was performed in R version 4.3 software (R Core Team, Vienna, Austria).

## RESULTS

A total of 615 patients at 6 sites were treated with FMT for rCDI. Four hundred seventy-three patients (77%) had follow-up data on CDI recurrence at 2 months. Twenty-five patients were then excluded for lack of non-CDI antibiotic exposure at 2 months, resulting in 448 participants for the primary analysis (Table 1). Patients had a median age of 66 (IQR, 49–75) years, with 54% (240/448) of the cohort aged ≥65 years (Table 1). Nineteen percent (82/448) of the cohort was immunocompromised and 34% (147/448) had been hospitalized previously for CDI (Table 1).

**Table 1.** Patient Demographics

Characteristic	No. (%) (n = 448)
Age, y, median (IQR)	66 (49–75)
Age ≥65 y	240 (54)
Female sex	330 (74)
White race	418 (94)
Immunocompromised	82 (19)
PPI usage	112 (25)
Inflammatory bowel disease	48 (11)
Hemodialysis use	12 (2.7)
Decompensated liver disease	8 (1.8)
Cognitive impairment	32 (7.1)
Neuromuscular impairment	55 (12)
Prior CDI hospitalization^a^	147 (34)
Antibiotic exposure prior to index CDI^b^	333 (76)
No.of rCDI episodes in prior year	
None	29 (6.5)
1	44 (9.9)
2	149 (34)
3	111 (25)
≥4	111 (25)
Prior CDI therapy^c^	
Metronidazole	90 (21)
Vancomycin, 10–14 d	392 (88)
Vancomycin tapered course	306 (68)
Fidaxomicin	180 (40)
Rifaximin	13 (2.9)
Bezlotoxumab	22 (4.9)
Prior FMT	82 (19)
FMT route	
Colonoscopic administration	162 (35)
Capsule administration	280 (63)
Other or mixed administration^d^	6 (1.3)

Data are presented as No. (%) unless otherwise indicated. Individuals with missing data were excluded from the percentage calculations.

Abbreviations: CDI, *Clostridioides difficile* infection; FMT, fecal microbiota transplantation; IQR, interquartile range; PPI, proton pump inhibitor; rCDI, recurrent *Clostridioides difficile* infection.

^a^Prior CDI hospitalization includes provider-reported assessment of CDI-related hospitalization (inclusive of hospital-onset CDI or admitted for treatment for CDI) at any point.

^b^Antibiotic exposure assessed by treating provider as an identifiable antibiotic administration preceding the initial CDI event that was considered contributory to CDI.

^c^Prior CDI therapy includes all previously received treatments for CDI.

^d^Other or mixed administration route included both colonoscopic and capsule administration, or liquid administration through a gastrointestinal tube.

Eleven percent (49/448) of patients were exposed to non-CDI antibiotics within 2 months of FMT. The indication for the non-CDI antibiotic administration in the first 2 months post-FMT was recorded in 45 of 49 cases. The most commonly reported indications for antibiotic administration were urinary tract infections (33% [15/45]), respiratory infections (16% [7/45]), and procedural prophylaxis (9% [4/45]). Information regarding specific antibiotic class exposure during the first 2 months post-FMT was collected when available (Supplementary Table 1).

### Non-CDI Antibiotic Exposure Risk Factors

Unlike other risk factors for CDI recurrence, the administration of non-CDI antibiotics post-FMT is a potentially modifiable risk factor. Therefore, we explored baseline factors that were associated with non-CDI antibiotic exposure within 2 months of FMT administration. The results of the univariate and multivariate analysis are presented in Table 2. Immunocompromised status, multiple courses of non-CDI antibiotics prior to FMT, and prior CDI hospitalization were all independently significantly associated with post-FMT non-CDI antibiotic exposure within 2 months.

**Table 2.** Associations of Baseline Characteristics on Non–*Clostridioides difficile* Infection Antibiotic Exposure Within 2 Months of Fecal Microbiota Transplantation

Characteristic	Univariate Analysis		Multivariate Analysis	
	OR (95% CI)	*P* Value	OR (95% CI)	*P* Value
Age	.99 (.98–1.01)	.7	.99 (.98–1.01)	.8
Female sex	.57 (.31–1.1)	.08	.59 (.31–1.1)	.1
Immunocompromised	2.7 (1.4–5.1)	**.002**	2.2 (1.1–4.4)	**.02**
Inflammatory bowel disease	.94 (.35–2.5)	.9	…	
Antibiotic trigger for index CDI	1.7 (.78–3.8)	.2	…	
PPI use	.64 (.3–1.4)	.3	…	
Hemodialysis use	2.8 (.74–11)	.1	…	
Decompensated liver disease	2.8 (.54–14)	.2	…	
Cognitive impairment	1.2 (.40–3.5)	.8	…	
≥3 non-CDI antibiotic courses pre-FMT	3.4 (1.6–7.4)	**.002**	3.1 (1.4–6.8)	**.006**
Neuromuscular impairment	2.0 (.94–4.3)	.07	…	
Prior CDI hospitalization	2.3 (1.3–4.2)	**.007**	2.0 (1.1–3.8)	**.02**
FMT route	1.0 (.54–1.9)	.9	…	
≥2 CDI recurrences pre-FMT	1.5 (.6–3.6)	.4	…	

Bold values indicate *P* value <.05.Abbreviations: CDI, *Clostridioides difficile* infection; CI, confidence interval; FMT, fecal microbiota transplantation; OR, odds ratio; PPI, proton pump inhibitor.

## DISCUSSION

In this study, we found that individuals who are immunocompromised, have multiple prior non-CDI antibiotics, or were previously hospitalized for CDI are at risk for non-CDI antibiotic administration within 2 months of FMT. Our findings highlight the importance of appropriate and judicious use of antibiotics, particularly post-FMT as antibiotic use is associated with CDI recurrence [14, 26].

Antibiotic stewardship is critical in managing CDI. An estimated 30% of outpatient antibiotic prescriptions may be inappropriate [28]. In certain circumstances such as dental prophylaxis, almost 80% of antibiotics administered may not be indicated. Even when appropriate, the selection and duration of antibiotics is frequently inappropriate, favoring broader-spectrum antibiotic selection with longer durations than recommended in guidelines [29]. As our data demonstrate that non-CDI antibiotic administration is associated with a 4-fold increase in the odds of CDI recurrence post-FMT, intervening on inappropriate antibiotic use is of critical importance to reduce subsequent CDI following FMT.

In addition to the appropriate use of antibiotics, practical strategies exist to mitigate the effect of non-CDI antibiotics on CDI recurrence post-FMT, although rigorous randomized controlled data in this area are lacking. These strategies include extending suppressive *C difficile* antibiotics (typically low-dose oral vancomycin) while delaying FMT, selection of antibiotics that minimally impact the colonic microbiota, or use of bezlotoxumab (with or without FMT). For example, patients who are transiently immunosuppressed (eg, undergoing a defined cycle of chemotherapy for cancer), have a planned surgical procedure, or are being worked up for a cause of recurrent infections (eg, urinary tract infection [UTI]) could be considered for an extended course of low-dose oral vancomycin with a plan for a delayed FMT. Some patients with uncomplicated UTIs could be administered aminoglycoside antibiotics, which have minimal impact on the colonic microbiota [30]. Similarly, the use of antibiotics with a lower risk of dysbiosis (eg, intravenous vancomycin) for surgical site infection prophylaxis could be considered in those with a high-risk profile for CDI. Providers administering FMT for prevention of CDI recurrence should be cognizant of the need for future antibiotics and develop an individualized plan to mitigate the impact of antibiotic use post-FMT. The efficacy of these suggested strategies is unclear and they come with potential trade-offs (eg, cost).

To that end, we identified risk factors for antibiotic administration within 2 months of FMT. We found that individuals who are immunocompromised, received multiple courses of non-CDI antibiotics, or were previously hospitalized for CDI are at an increased risk for non-CDI antibiotic administration within 2 months of FMT. This finding is in line with previously described higher risk for antibiotic exposure in immunocompromised patients [31]. Assessment of these risk factors along with understanding the initial triggering event for CDI can help develop a comprehensive plan to prevent subsequent CDI episodes. Of our identified risk factors, administration of multiple (≥3) courses of non-CDI antibiotics in the 12 months preceding FMT was associated with a 3-times increase in the odds of non-CDI antibiotic use post-FMT. As UTIs were the most common cause of post-FMT non-CDI antibiotic use, we hypothesize that recurrent UTIs was a major contributor of rCDI in our patient population. Employing one of the strategies discussed above could be considered along with an evaluation for preventing recurrent UTIs. Further research into the appropriateness of use of antibiotics in treating UTIs in this patient population would be warranted to assess to what degree this effect may be modifiable.

Bezlotoxumab could be a useful option for individuals who are likely to receive antibiotics post-FMT. Bezlotoxumab is a human monoclonal antibody available as an intravenous infusion that binds and neutralizes *C difficile* toxin B and reduces the risk of rCDI during treatment for primary or recurrent CDI with antibiotic therapy [32, 33]. Bezlotoxumab provides temporary passive immunity against CDI that, in theory, should be independent of antibiotic exposure. In individuals with rCDI at risk for subsequent antibiotic use, bezlotoxumab could be considered as an alternative to FMT or given in conjunction with FMT. While a combination of bezlotoxumab and FMT was not shown to have clear benefit in patients with rCDI and inflammatory bowel disease, there are case reports of its successful concomitant use for patients with rCDI [34, 35]. However, the availability of bezlotoxumab may be limited as Merck announced its discontinuation on 31 January 2025 [36].

Our cohort has several strengths including the large size and the real-world representation of patients treated with FMT in both academic and community settings. These patients often have comorbidities or risk factors that would otherwise exclude them from randomized clinical trials [37]. Thus, our findings are likely generalizable to most practitioners treating CDI patients. This study also has some important limitations. Data were collected prospectively through centralized case report forms, but this was performed in conjunction with clinical care, not separate research visits. As such, some data were extracted from medical records and not patient encounters. Despite extensive attempts to minimize missing data, some outcome data remained incomplete, particularly after 2 months of follow-up time. Another limitation was the classification of CDI recurrence. Although all participants had both laboratory testing consistent with CDI and initiation of anti-CDI antibiotics, we do not have granular detail on the type of laboratory testing used to support the diagnosis, potentially leading to some misclassification of CDI recurrence. Additionally, some data were collected as free-text, specifically the indication for non-CDI antibiotic administration. The CDI risk varies with antibiotic type and duration; however, we are unable to assess this level of detail. Some of our risk factors assessed were not validated. For example, classification of cognitive impairment or decompensated liver disease can vary between providers. Thus, misclassification of potential risk factors could have occurred. No formal sample size and power testing was performed and as such the study may be underpowered to detect smaller risks. Although no differences were seen in the patient population and primary outcome between sites (data not shown), it is possible that site-specific antibiotic stewardship practices could have influenced our results. Finally, availability of FMT has been recently severely restricted in the United States by the most recent FDA guidance, which no longer allows nonprofit stool bank operations. However, our findings likely extrapolate to existing commercial fecal microbiota–based therapies that are FDA approved.

In conclusion, we confirmed that non-CDI antibiotic exposure is a risk factor for CDI recurrence post-FMT. Additionally, we identify that immunocompromise, multiple prior non-CDI antibiotic courses, and prior hospitalization for CDI increase the risk for non-CDI antibiotic exposure post-FMT. These factors should prompt providers to consider alternative strategies to prevent CDI recurrence such as delaying FMT into the treatment strategy.

## Supplementary Material

ofaf130_Supplementary_Data

## Supplementary Data

Supplementary materials are available at *Open Forum Infectious Diseases* online. Consisting of data provided by the authors to benefit the reader, the posted materials are not copyedited and are the sole responsibility of the authors, so questions or comments should be addressed to the corresponding author.

## Notes

***Author contributions.*** W. H. was responsible for contributing to statistical analysis, interpretation of results, and writing and critical review of the manuscript. B. V. was responsible for study concept and design, contributing to statistical analysis, interpretation of results, and writing and critical review of the manuscript. M. F., A. K., J. R. A., and C. R. K. were responsible for interpretation of results, data acquisition, and critical review and editing of the manuscript.

***Patient consent.*** The protocol was approved by the University of Minnesota Institutional Review Board.

## References

[1] Leffler DA, Lamont JT. *Clostridium difficile* infection. N Engl J Med 2015; 372:1539–48. 25875259 10.1056/NEJMra1403772

[2] Bouza E . Consequences of *Clostridium difficile* infection: understanding the healthcare burden. Clin Microbiol Infect 2012; 18:5–12. 10.1111/1469-0691.12064 23121549

[3] Fekety R, McFarland LV, Surawicz CM, Greenberg RN, Elmer GW, Mulligan ME. Recurrent *Clostridium difficile* diarrhea: characteristics of and risk factors for patients enrolled in a prospective, randomized, double-blinded trial. Clin Infect Dis 1997; 24:324–33. 9114180 10.1093/clinids/24.3.324

[4] Kelly CP, LaMont JT. *Clostridium difficile*—more difficult than ever. N Engl J Med 2008; 359:1932–40. 18971494 10.1056/NEJMra0707500

[5] van Nood E, Vrieze A, Nieuwdorp M, et al Duodenal infusion of donor feces for recurrent *Clostridium difficile*. N Engl J Med 2013; 368:407–15. 23323867 10.1056/NEJMoa1205037

[6] Wei S, Bahl MI, Baunwall SMD, Dahlerup JF, Hvas CL, Licht TR. Gut microbiota differs between treatment outcomes early after fecal microbiota transplantation against recurrent *Clostridioides difficile* infection. Gut Microbes 2022; 14:2084306. 36519447 10.1080/19490976.2022.2084306 PMC9176232

[7] Ianiro G, Punčochář M, Karcher N, et al Variability of strain engraftment and predictability of microbiome composition after fecal microbiota transplantation across different diseases. Nat Med 2022; 28:1913–23. 36109637 10.1038/s41591-022-01964-3 PMC9499858

[8] Hvas CL, Dahl Jørgensen SM, Jørgensen SP, et al Fecal microbiota transplantation is superior to fidaxomicin for treatment of recurrent *Clostridium difficile* infection. Gastroenterology 2019; 156:1324–32.e3. 30610862 10.1053/j.gastro.2018.12.019

[9] Kao D, Roach B, Silva M, et al Effect of oral capsule- vs colonoscopy-delivered fecal microbiota transplantation on recurrent *Clostridium difficile* infection: a randomized clinical trial. JAMA 2017; 318:1985–93. 29183074 10.1001/jama.2017.17077 PMC5820695

[10] Du C, Luo Y, Walsh S, Grinspan A. Oral fecal microbiota transplant capsules are safe and effective for recurrent *Clostridioides difficile* infection: a systematic review and meta-analysis. J Clin Gastroenterol 2021; 55:300–8. 33471490 10.1097/MCG.0000000000001495

[11] Kassam Z, Lee CH, Yuan Y, Hunt RH. Fecal microbiota transplantation for *Clostridium difficile* infection: systematic review and meta-analysis. Am J Gastroenterol 2013; 108:500–8. 23511459 10.1038/ajg.2013.59

[12] Quraishi MN, Widlak M, Bhala N, et al Systematic review with meta-analysis: the efficacy of faecal microbiota transplantation for the treatment of recurrent and refractory *Clostridium difficile* infection. Aliment Pharmacol Ther 2017; 46:479–93. 28707337 10.1111/apt.14201

[13] Minkoff NZ, Aslam S, Medina M, et al Fecal microbiota transplantation for the treatment of recurrent *Clostridioides difficile* (*Clostridium difficile*). Cochrane Database Syst Rev 2023; 4:CD013871. 37096495 10.1002/14651858.CD013871.pub2 PMC10125800

[14] Vaughn BP, Fischer M, Kelly CR, et al Effectiveness and safety of colonic and capsule fecal microbiota transplantation for recurrent *Clostridioides difficile* infection. Clin Gastroenterol Hepatol 2023; 21:1330–7.e2. 36126907 10.1016/j.cgh.2022.09.008

[15] Johnson S, Lavergne V, Skinner AM, et al Clinical practice guideline by the Infectious Diseases Society of America (IDSA) and Society for Healthcare Epidemiology of America (SHEA): 2021 focused update guidelines on management of *Clostridioides difficile* infection in adults. Clin Infect Dis 2021; 73:e1029–44. 34164674 10.1093/cid/ciab549

[16] Kelly CR, Fischer M, Allegretti JR, et al ACG clinical guidelines: prevention, diagnosis, and treatment of *Clostridioides difficile* infections. Am J Gastroenterol 2021; 116:1124. 34003176 10.14309/ajg.0000000000001278

[17] Di Bella S, Sanson G, Monticelli J, et al *Clostridioides difficile* infection: history, epidemiology, risk factors, prevention, clinical manifestations, treatment, and future options. Clin Microbiol Rev 2024; 37:e0013523. 38421181 10.1128/cmr.00135-23 PMC11324037

[18] Negrut N, Bungau S, Behl T, et al Risk factors associated with recurrent *Clostridioides difficile* infection. Healthcare (Basel) 2020; 8:352. 32967323 10.3390/healthcare8030352 PMC7551610

[19] Deshpande A, Pasupuleti V, Thota P, et al Risk factors for recurrent *Clostridium difficile* infection: a systematic review and meta-analysis. Infect Control Hosp Epidemiol 2015; 36:452–60. 25626326 10.1017/ice.2014.88

[20] Eeuwijk J, Ferreira G, Yarzabal JP, Robert-Du Ry van Beest Holle M . A systematic literature review on risk factors for and timing of *Clostridioides difficile* infection in the United States. Infect Dis Ther 2024; 13:273–98. 38349594 10.1007/s40121-024-00919-0 PMC10904710

[21] Chakra CNA, Pepin J, Sirard S, Valiquette L. Risk factors for recurrence, complications and mortality in *Clostridium difficile* infection: a systematic review. PLoS One 2014; 9:e98400. 24897375 10.1371/journal.pone.0098400 PMC4045753

[22] Garey KW, Sethi S, Yadav Y, DuPont HL. Meta-analysis to assess risk factors for recurrent *Clostridium difficile* infection. J Hosp Infect 2008; 70:298–304. 18951661 10.1016/j.jhin.2008.08.012

[23] Eze P, Balsells E, Kyaw MH, Nair H. Risk factors for *Clostridium difficile* infections—an overview of the evidence base and challenges in data synthesis. J Glob Health 2017; 7:010417. 28607673 10.7189/jogh.07.010417 PMC5460399

[24] Kwok CS, Arthur AK, Anibueze CI, Singh S, Cavallazzi R, Loke YK. Risk of *Clostridium difficile* infection with acid suppressing drugs and antibiotics: meta-analysis. Am J Gastroenterol 2012; 107:1011–9. 22525304 10.1038/ajg.2012.108

[25] Thongprayoon C, Cheungpasitporn W, Phatharacharukul P, et al Chronic kidney disease and end-stage renal disease are risk factors for poor outcomes of *Clostridium difficile* infection: a systematic review and meta-analysis. Int J Clin Pract 2015; 69:998–1006. 26147121 10.1111/ijcp.12672 PMC4552593

[26] Allegretti JR, Kao D, Sitko J, Fischer M, Kassam Z. Early antibiotic use after fecal microbiota transplantation increases risk of treatment failure. Clin Infect Dis 2018; 66:134–5. 29020157 10.1093/cid/cix684

[27] Youssef J, Novosad S, Winthrop K. Infection risk and safety of corticosteroid use. Rheum Dis Clin North Am 2016; 42:157–76. 26611557 10.1016/j.rdc.2015.08.004 PMC4751577

[28] Fleming-Dutra KE, Hersh AL, Shapiro DJ, et al Prevalence of inappropriate antibiotic prescriptions among US ambulatory care visits, 2010–2011. JAMA 2016; 315:1864–73. 27139059 10.1001/jama.2016.4151

[29] Durkin MJ, Keller M, Butler AM, et al An assessment of inappropriate antibiotic use and guideline adherence for uncomplicated urinary tract infections. Open Forum Infect Dis 2018; 5:ofy198. 30191156 10.1093/ofid/ofy198 PMC6121225

[30] Staley C, Vaughn BP, Graiziger CT, Sadowsky MJ, Khoruts A. Gut-sparing treatment of urinary tract infection in patients at high risk of *Clostridium difficile* infection. J Antimicrob Chemother 2017; 72:522–8. 27999027 10.1093/jac/dkw499 PMC6075516

[31] Hand J, Imlay H. Antimicrobial stewardship in immunocompromised patients: current state and future opportunities. Infect Dis Clin North Am 2023; 37:823–51. 37741735 10.1016/j.idc.2023.08.002

[32] Gerding DN, Kelly CP, Rahav G, et al Bezlotoxumab for prevention of recurrent *Clostridium difficile* infection in patients at increased risk for recurrence. Clin Infect Dis 2018; 67:649–56. 29538686 10.1093/cid/ciy171 PMC6093994

[33] Wilcox MH, Gerding DN, Poxton IR, et al Bezlotoxumab for prevention of recurrent *Clostridium difficile* infection. N Engl J Med 2017; 376:305–17. 28121498 10.1056/NEJMoa1602615

[34] Allegretti JR, Kelly CR, Grinspan A, Mullish BH, Kassam Z, Fischer M. Outcomes of fecal microbiota transplantation in patients with inflammatory bowel diseases and recurrent *Clostridioides difficile* infection. Gastroenterology 2020; 159:1982–4. 32738249 10.1053/j.gastro.2020.07.045

[35] Kaako A, Al-Amer M, Abdeen Y. Bezlotoxumab use as adjunctive therapy with the third fecal microbiota transplant in refractory recurrent *Clostridium difficile* colitis; a case report and concise literature review. Anaerobe 2019; 55:112–6. 30521856 10.1016/j.anaerobe.2018.11.010

[36] Ernst D . C. *difficile* prevention therapy Zinplava discontinued. Medical Professionals Reference. 2025. Available at: https://www.empr.com/news/c-difficile-prevention-therapy-zinplava-discontinued/. Accessed 3 February 2025.

[37] Kelly CR, Fischer M, Grinspan A, Allegretti JR. Patients eligible for trials of microbe-based therapeutics do not represent the population with recurrent *Clostridioides difficile* infection. Clin Gastroenterol Hepatol 2020; 18:1099–101. 31254675 10.1016/j.cgh.2019.06.034

