# Supplementary material for: Risk Factors for Antibiotic Exposure Post–Fecal Microbiota Transplantation for Recurrent Clostridioides difficile Infection: A Prospective Multicenter Observational Study
Source: Open Forum Infect Dis. 2025 Mar 7;12(3):ofaf130. doi: 10.1093/ofid/ofaf130 (PMC11913780; doi:10.1093/ofid/ofaf130)
Supplement: ofaf130_Supplementary_Data [file ofaf130_supplementary_data.docx]

Supplemental Table 1: Antibiotic Exposure During First Two Months Post FMT

| Antibiotic Class | n = 49 (%) |
| --- | --- |
| Beta-lactam | 13 (27) |
| Glycopeptide (intravenous) | 10 (20) |
| Aminoglycoside | 6 (12) |
| Fluoroquinolone | 6 (12) |
| Tetracycline | 6 (12) |
| Macrolide | 5 (10) |
| Sulfonamide | 5 (10) |
| Nitroimidazole | 4 (8) |
| Lincosamide | 1 (2) |
| Nitrofuran | 1 (2) |
